# Determining the effective dose of esketamine combined with propofol for painless hysteroscopy: a prospective dose-finding study

**DOI:** 10.3389/fphar.2024.1419732

**Published:** 2024-12-16

**Authors:** Zhimin Sheng, Xiang Liu, Kang Lin, Jie Liu, Junqin Mao, Xiaowei Qian

**Affiliations:** ^1^ Department of Anesthesiology, Wenling Maternity and Child Healthcare Hospital, Taizhou, China; ^2^ Department of Anesthesiology, Women’s Hospital, Zhejiang University School of Medicine, Hangzhou, China; ^3^ Department of Anesthesiology, Wenling First People’s Hospital (The Affiliated Wenling Hospital of Wenzhou Medical University), Taizhou, China

**Keywords:** dose-response, dixon’s up and down method, esketamine-propofol combination, 95% effective dose (ED95), painless hysteroscopy

## Abstract

**Background:**

The combination of esketamine and propofol has become a common choice for total intravenous anesthesia in hysteroscopic procedures. However, the optimal effective dose has not yet been determined. The aim of this study was to determine the median effective dose (ED_50_) and 95% effective dose (ED_95_) of esketamine compounded with propofol for painless hysteroscopy.

**Methods:**

A total of 40 patients aged 20–60 years and scheduled for painless hysteroscopy under intravenous anesthesia were recruited, and a total of 31 patients were enrolled for the final analysis. Using the Dixon’s up and down method, an initial dose of 0.5 mg/kg esketamine was administered intravenously before surgery, and after 1 min, it was followed by 2 mg/kg of propofol. If the hysteroscopy failed (a positive reaction) [defined as inadequate cervical dilatation, patient body movements interfering with surgical procedures during hysteroscopy placement, frowning, or Ramsay Sedation Scale (RSS) score <5 within 5 min], the subsequent patient’s esketamine dosage was increased by 0.1 mg/kg. Conversely (a negative reaction), the dosage was decreased by 0.1 mg/kg. The test was not stopped until at least 7 crossovers occurred. The perioperative adverse events of each patient were recorded. The ED_50_ and ED_95_ with 95% confidence intervals (CIs) were estimated using probit regression.

**Results:**

The ED_50_ and ED_95_ with 95% (CIs) of esketamine in patients were 0.287 (0.220–0.342) mg/kg and 0.429 (0.365–0.705) mg/kg, respectively. No serious adverse events were observed in any patients.

**Conclusion:**

A dose of 0.429 mg/kg esketamine combined with propofol is recommended for painless hysteroscopy anesthesia, as it enhances anesthesia and postoperative analgesia efficacy without significant adverse reactions. However, potential risks associated with this dosage should be carefully considered in clinical practice.

**Clinical Trial Registration:**

https://www.chictr.org.cn/index.html, identifier ChiCTR2300075564.

## 1 Introduction

With the development of minimally invasive technique and assisted reproductive technologies, hysteroscopy has become widely used in gynecological examinations and treatments. Its ability to visualize the entire uterine cavity and ensure quick recovery has led to its recognition as the “gold standard” for diagnosing and treating intrauterine pathologies (Vitale et al., 2020; Zargar et al., 2020). However, most patients are unable to tolerate the discomfort and intense pain caused by cervical canal dilation and endometrial cutting without analgesia and sedation (Ahmad et al., 2017).

Propofol is widely used for the induction and maintenance of anesthesia in hysteroscopic surgery due to its rapid onset, short duration of action, and limited side effects (Zhang et al., 2018). However, the analgesic effect of propofol is notably insufficient and usually requires adjunctive analgesic agents (Godambe et al., 2003).

Esketamine, an S-enantiomer of ketamine, is a non-competitive N-methyl-D-aspartate (NMDA) receptor antagonist (Zanos and Gould, 2018). It stands out for its mild respiratory depression, complete recovery, and rapid onset of action, providing both analgesic and sedative effects with fewer psychological side effects compared to ketamine (Pfenninger et al., 2002). Furthermore, its sympathomimetic properties offer a counterbalance to the hemodynamic inhibition of propofol, thus reducing the risk of respiratory depression and cardiovascular issues during sedation (Eberl et al., 2017; Chen et al., 2022). Prior research has indicated that 0.5 mg/kg of esketamine combined with propofol for hysteroscopy anesthesialowers the intraoperative propofol dosage and minimizes adverse effects on the circulatory and respiratory systems (Wang et al., 2023).

Although esketamine is becoming increasingly popular in hysteroscopy, the optimal effective dose for the combination of esketamine and propofol has not yet been determined. Therefore, this study aims to primarily investigate the median effective dose (ED_50_) and 95% effective dose (ED_95_) of esketamine compounded with propofol for painless hysteroscopy.

## 2 Materials and methods

### 2.1 Design and study subjects

This dose-finding study was conducted from September 2023 to February 2024 at the Wenling Maternity and Child Health Care Hospital, Taizhou, China, and received approval from the Ethical Committee (Approval No. 2023-IRB-102). The protocol was registered in the Chinese Clinical Trial Registry (www.chictr.org.cn; No. ChiCTR2300075564) on 8 September 2023. Written informed consent was signed by all subjects before enrollment in this study. The study was conducted in accordance with the Declaration of Helsinki and the Consolidated Standards of Reporting Trials (CONSORT).

Patients aged 20–60 years with American Society of Anesthesiologists (ASA) physical status of I or II, who were scheduled for painless hysteroscopy under intravenous anesthesia in our institution were recruited. Patients were excluded if they had a history of the following conditions: (1) Allergy to propofol or esketamine; (2) Chronic use of opioids, tranquillizers, or antidepressants; (3) Combined with cardiovascular and respiratory diseases; (4) Dysfunction of liver and kidney; (5) Hypertension, diabetes, hyperthyroidism, increased intraocular or intracranial pressure; (6) Have a history of mental illness or psychological issues; (7) Congenital cervical canal or uterine malformation with severe intrauterine adhesion that make hysteroscopy difficult to implement.

As this was an adaptive clinical trial, the sample size was not calculated beforehand, allowing for flexibility according to specific criteria with a minimum seven-crossover rule to terminate subject inclusion. However, simulation studies have demonstrated that enrolling 20 to 40 subjects would provide stable estimates of the ED_50_ using the Dixon’s up-and-down method (Pace and Stylianou, 2007). Additionally, to account for potential dropouts and to achieve narrower confidence intervals, we decided to include at least 30 subjects in this trial.

### 2.2 Study protocol

All patients were required to food-fasted for at least 8 h and clear liquids-fasted for 2 h prior to anesthesia. No premedication was administered before surgery. Routine monitoring including noninvasive blood pressure, pulse oxygen saturation (SpO_2_), electrocardiography, respiratory rate and heart rate (HR) was performed when the patient attended the operating room. Oxygen with a venturi mask at a rate of 5 L/min was inhaled to all patients. An 18-gauge intravenous cannula was inserted into the peripheral vein before anesthesia induction. On the day of surgery, all doses of esketamine were diluted with normal saline to 10 mL inidentical syringes and labelled as study drugs by a nurse anesthetist, who was not involved in the study. The syringes were then handed over to the anesthesiologist responsible for recording the intraoperative data and was blinded to the dosage administered to the patient.

The Dixon’s up and down method was adopted in this study. Esketamine (Jiangsu Hengrui Pharmaceuticals Co. Ltd., China) was administered intravenously prior to the surgery, starting with an initial dose of 0.5 mg/kg based on preliminary experiment and previously published studies (Wang et al., 2023; Zheng et al., 2022). At 1 min after the onset, followed by 2 mg/kg of propofol (Aspen Pharma Co. Ltd., Ireland) intravenously at a constant speed within 30–60 s, and then maintained at 6 mg/kg/h. The dosage of propofol was determined based on previous studies and our clinical experience, which demonstrated its effectiveness for induction in similar settings (Yu et al., 2019; Zhong et al., 2022). Hysteroscopy was performed by an experienced gynecologist after the patient lost consciousness and the eyelash reflex. Additional propofol 0.5 mg/kg was added intravenously if the hysteroscopy failed (a positive reaction) [defined as inadequate cervical dilatation, patient body movements interfering with surgical procedures during hysteroscopy placement, frowning, or Ramsay Sedation Scale (RSS) score <5 within 5 min], and the top-up interval was greater than 1 min until the required depth of anesthesia for the procedure was achieved. In this case, the next patient’s esketamine dosage was increased by 0.1 mg/kg. Conversely (a negative reaction), the dosage was decreased by 0.1 mg/kg.

The RSS score was assessed on a 6-point scale: 1 point: Awake, anxious or agitated; 2 point: Awake, tranquil, oriented and cooperative; 3 point: Awake, drowsy, only responding to commands; 4 point: Asleep, brisk response to loud auditory stimulus or taps; 5 point: Asleep, sluggish response to loud auditory stimulus or taps; 6 point: Asleep, no response to stimulation (Ramsay et al., 1974).

Patients were divided into two groups based on their responses: Group P (positive reaction) and Group N (negative reaction), determined by the adequacy of cervical dilation, body movement, and RSS score during hysteroscopy.

Ephedrine (5–10 mg) was given as needed for hypotension [systolic blood pressure (SBP) decreases >20% of baseline)]. Bradycardia was treated with atropine (0.3–0.5 mg). Respiratory depression (SpO_2_ below 90%) was managed with oxygen assistance via a facial mask.

The main observation was the success rate of the procedure, defined as the patient not exhibiting body movements interfering with surgical procedures during hysteroscopy placement, RSS score ≥5 within 5 min and no rescue doses were required. Secondary observations included the dose of esketamine, initial and total doses of propofol, the operation duration, recovery time from anesthesia, visual analog scores (VAS) at 2 h postoperatively (0 = painless, 10 = severe pain) and the incidence of various side effects (such as respiratory depression, hypotension, hypertension, nausea, vomiting, bradycardia, visual disturbance, and dizziness during the awakening period).

### 2.3 Statistical analysis

Data analyses were conducted using IBM SPSS 22.0 for windows (Corp, Armonk, NY) and GraphPad Prism 8.0.2 (GraphPad Software, San Diego, CA, United States). Normally distributed data were compared between two groups using the independent-samples Student’s t-test and presented as means ± standard deviation (SD). For non-normally distributed data, comparison was made using the Mann-Whitney *U*-test and presented as median [interquartile range]. Categorical data were expressed as percentages (n, %) and analyzed using Chi-square tests.

The dose-response relationship was analyzed using probit regression, which estimated the doses required to achieve ED_50_ and ED_95_ probability of the desired clinical effect when esketamine was combined with propofol. Confidence intervals (CIs) for ED_50_ and ED_95_ were calculated to ensure precision and reliability of these estimates. A *p*-value < 0.05 was deemed statistically significant.

## 3 Results

**FIGURE 1:**
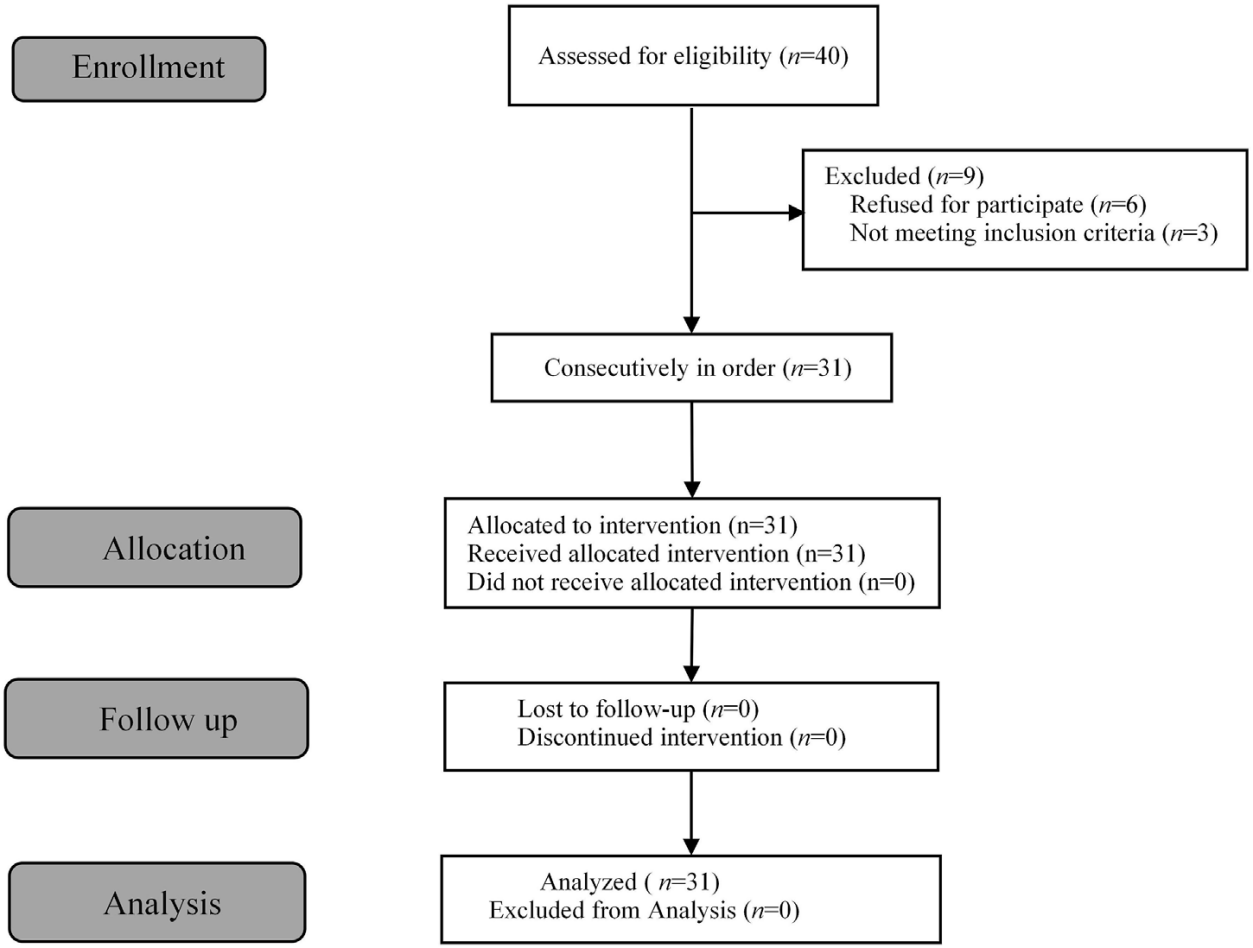
Flow diagram of the study.

**FIGURE 2:**
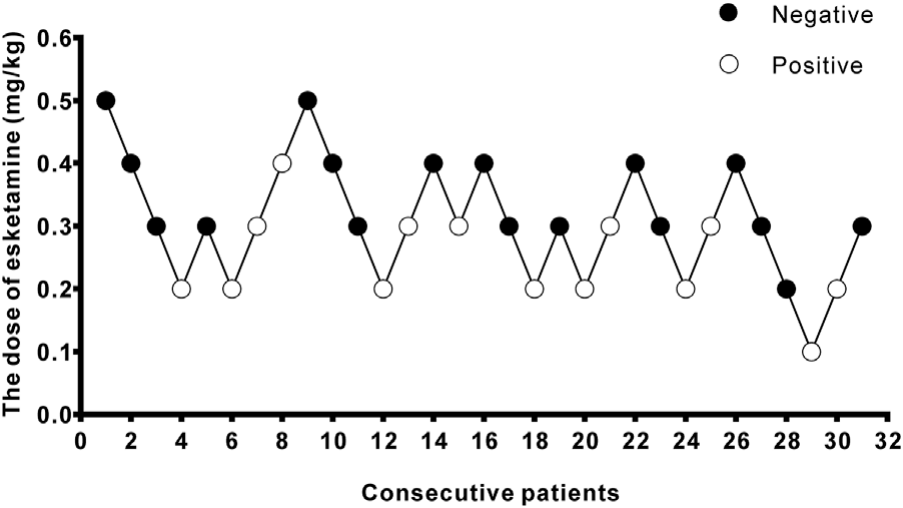
Stepwise dose adjustment of esketamine for painless hysteroscopy using Dixon’s up and down method. Solid circles represent negative reactions; hollow circles represent positive reactions.

**FIGURE 3:**
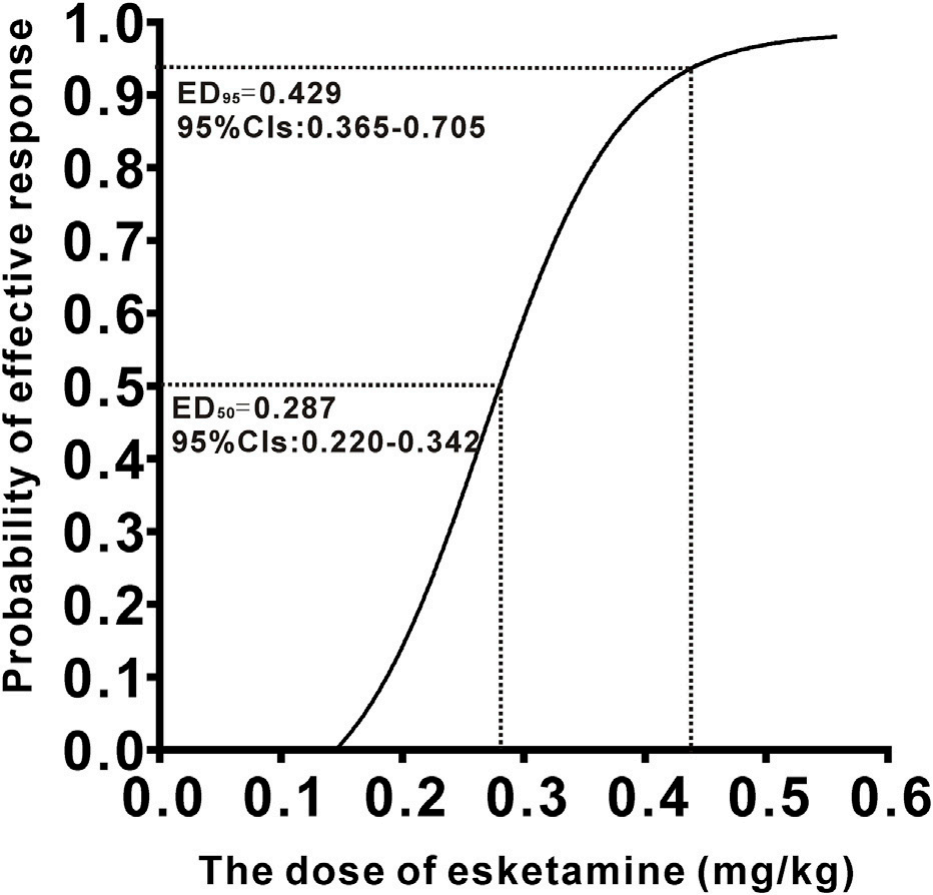
Dose–response curve of esketamine for painless hysteroscopic surgery.

A total of 40 patients were recruited in this study, and 9 patients were excluded (Figure 1). The demographic characteristics of the participants in both groups are summarized in Table 1. The age, weight, height, and body mass index were similar between the Group P and Group N. The perioperative profiles of the patients are presented in Table 2. There was no statistically significant difference in perioperative profiles between the two groups, except for the dose of esketamine, total dose of propofol and time to recovery. No adverse effects such as nausea and vomiting, hypotension, bradycardia, visual disturbance, and dizziness were observed during the perioperative period. Only two patients developed transient respiratory depression, and one patient developed reactive hypertension in Group N. One patient had a VAS score greater than 3 points 2 h after surgery in the Group P. The sequential doses of esketamine co-administered with propofol for painless hysteroscopy are shown in Figure 2. The probability unit regression equation fitted based on probit regression analysis was: probit (P) = −3.306 + 11.529 × esketamine dose. The Pearson goodness-of-fit chi-square statistic χ^2^ = 0.434 (*p* = 0.933) confirmed that the model fully fitted the data. The derived dose-response curves of esketamine for painless hysteroscopy showed that the values of ED_50_ and ED_95_ were 0.287 (0.220–0.342) mg/kg and 0.429 (0.365–0.705) mg/kg, respectively (Figure 3).

**TABLE 1:** Demographic data.

	Group P (*n* = 14)	Group N (*n* = 17)	p-value
Age (years)	36.1 ± 9.1	39.9 ± 8.9	0.260
Height (cm)	159.4 ± 4.8	157.4 ± 6.2	0.327
Weight (kg)	57.9 ± 7.7	57.7 ± 6.7	0.930
BMI (kg/cm^2^)	23.1 ± 2.7	23.3 ± 2.4	0.831

Data are presented as mean ± SD (standard deviation).

BMI, body mass index.

**TABLE 2:** Perioperative profiles between the two groups of patients.

	Group P (*n* = 14)	Group N (*n* = 17)	p-value
Dose of esketamine (mg)	14.3 ± 5.8	20.5 ± 6.0	0.007^*^
Initial dose of propofol (mg)	116.6 ± 15.3	115.5 ± 13.4	0.822
Total dose of propofol (mg)	176.2 ± 43.6	132.3 ± 24.8	0.001^*^
Operation duration (min)	14.6 ± 3.9	14.5 ± 3.7	0.942
Time to recovery (min)	7.3 ± 1.4	6.1 ± 1.2	0.018*
VAS pain score >3 (points)	1 (7.1%)	0 (0.0%)	0.263
Respiratory depression (n)	0 (0.0%)	2 (11.8%)	0.185
Nausea and vomiting (n)	0 (0.0%)	0 (0.0%)	—
Hypotension (n)	0 (0.0%)	0 (0.0%)	—
Hypertension (n)	0 (0.0%)	1 (5.9%)	0.356
Bradycardia (n)	0 (0.0%)	0 (0.0%)	—
Visual disturbance during awakening period (n)	0 (0.0%)	0 (0.0%)	—
Dizziness during awakening period (n)	0 (0.0%)	0 (0.0%)	—

Data are presented as mean ± SD, or number (%). Categorical data were analyzed using the Cochran–Armitage χ2 test for trend. Asterisks denote a statistical difference, two-sided *t*-test, *p < 0.05.

VAS, visual analogue scale.

## 4 Discussion

In this up-and-down sequential allocation dose-finding study, we determined that the ED_50_ and ED_95_ values of esketamine combined with propofol (2 mg/kg) for intravenous sedation in painless hysteroscopy were 0.287 (0.220–0.342) mg/kg and 0.429 (0.365–0.705) mg/kg, respectively.

As a novel anesthetic, esketamine exerts sedative and analgesic effects mainly by acting on certain sodium channels through NMDA receptors (Li et al., 2022). It also blocks sodium channels in the brainstem parasympathetic nerves, leading to sympathetic nerve excitation, increased cardiac output, and peripheral vascular resistance (Jang et al., 2020; Xue et al., 2020). These characteristics make it an ideal adjunct to propofol with less cardiopulmonary inhibition compared to other sedative anesthetics (Li et al., 2022).

Numerous studies have demonstrated that the combination of esketamine and propofol has advantages in endoscopic retrograde cholangiopancreatography and hysteroscopy anesthesia, such as reducing propofol dosage, having minimal impact on the circulation and respiratory systems of patients, and improving postoperative analgesia, which is consistent with our findings (Eberl et al., 2020; Wang et al., 2023).

The Dixon’s up and down method was adopted to determine the ED_50_ and ED_95_ of esketamine in this study, since it has the advantages of simplicity, effectiveness, and smaller sample size requirements compared to other methods. Based on previous research (Wang et al., 2023; Wang et al., 2019), the initial dose of esketamine was set at 0.5 mg/kg with a designed iso-differential concentration of 0.1 mg/kg. The test continued until at least 7 crossovers occurred, and an RSS score ≥5 was used as the criterion for effective sedation (Ramsay et al., 1974; Zhong et al., 2022).

It is important to highlight the common side effects of propofol, including circulatory and respiratory suppression and injection pain. In contrast to previous reports of significant hypotension with propofol alone (Zhang et al., 2018), our study demonstrated a lower incidence, likely due to the complementary pharmacological effects of the esketamine-propofol combination. Previous studies have indicated that esketamine’s sympathomimetic properties may help mitigate propofol-induced hypotension, while its vagal antagonism can reduce the incidence of bradycardia during hysteroscopy (Xue et al., 2020; Lei et al., 2021). In our study, only 2 out of 20 patients in Group N developed short-term respiratory depression, which was relieved quickly by the jaw thrust maneuver. This may be related to the sympathomimetic effect of esketamine, which enhances respiratory rate and attenuates respiratory inhibition caused by propofol. Additionally, esketamine can directly antagonize the spasmodic effect of histamine on bronchial smooth muscle, enhance the relaxation effect of catecholamines on bronchial smooth muscle, and improve lung compliance (Corssen et al., 1972). Injection pain is also a common complaint associated with propofol administration, typically attributed to its high lipid content and hyperosmolarity. While this side effect was not directly assessed in our study, previous research reported a significant reduction in propofol-induced injection pain when co-administered with esketamine (Xu et al., 2022).

No patients in our study experienced nausea and vomiting, visual disturbances, or dizziness during the perioperative period, which may be attributed to the counteracting effects of propofol’s anxiolytic and antiemetic properties on esketamine - induced emergence reactions (Willman and Andolfatto, 2007). Only one patient in Group P had a VAS score >3 at 2 h after surgery, indicating a strong analgesic effect of esketamine. Previous studies have also confirmed its beneficial role in postoperative analgesia without obvious adverse effects (Brinck et al., 2021; Bornemann-Cimenti et al., 2016).

The psychotomimetic side effects and cognitive function changes caused by esketamine remain significant concerns. Zheng et al. reported high incidences of visual dysfunction and emergency delirium in the 1 mg/kg esketamine group (Zheng et al., 2022), while the patients in this study did not experience such mental issues during the recovery period. Several explanations have been proposed for this discrepancy. First, our study exclusively focused on female patients who underwent painless hysteroscopy, while their study subjects were pediatric patients who underwent upper gastrointestinal endoscopy. Second, the relationship between psychiatric adverse reactions and esketamine dosage appears to be dose-dependent manner (Zhu et al., 2022). The highest dose group in the current study was 0.5 mg/kg, compared to 1 mg/kg in Zheng et al.'s work (Zheng et al., 2022), potentially accounting for the variance in outcomes. Third, it is also possible that varying doses of propofol used in the two studies weakened the psychogenic side effects of esketamine through different levels of activation of GABA receptors.

Several limitations of this study warrant attention. Firstly, according to previous studies, the initial dose of intravenous propofol in this study was set at 2 mg/kg (Yu et al., 2019; Zhong et al., 2022). It has been shown that the ED_50_ value of propofol decreased when co-administered with increasing doses of esketamine (Zheng et al., 2022; Hayes et al., 2018). Therefore, the equilibrium point between the optimal clinical effect and minimal adverse effects across varying doses of propofol and esketamine should be further explored. Secondly, we employed the RSS to assess the level of sedation in patients. However, compared to the Bispectral Index (BIS), it lacks objective criteria and exhibits low accuracy. BIS monitoring does not require testing the patient’s response to stimuli, which is particularly advantageous when it is not convenient to use verbal or physical stimuli to observe the patient’s response to determine the level of sedation. Thirdly, in clinical practice, sub-anesthetic doses of esketamine (0.1–0.3 mg/kg) are commonly used to minimize adverse reactions. However, the highest dose group of esketamine in this study was 0.5 mg/kg. Although no significant intraoperative agitation or delirium was observed, further investigation is required to assess the potential postoperative adverse effects associated with the recommended dose of 0.429 mg/kg. Fourthly, the small sample size inherent in Dixon’s up and down method may introduce selection bias; therefore, experimental results should be validated by a multi-center study with a larger sample size.

## 5 Conclusion

To sum up, our study determined that the ED_50_ and ED_95_ for esketamine combined with propofol in painless hysteroscopy anesthesia were 0.287 mg/kg and 0.429 mg/kg, respectively. While this dosing scheme demonstrated improved anesthesia and postoperative analgesic effectiveness in patients without significant adverse reactions in our study, caution should be exercised when applying the ED_95_ dose in clinical practice due to the potential risk of esketamine-induced adverse effects.

## Data Availability

The original contributions presented in the study are included in the article/supplementary material, further inquiries can be directed to the corresponding author.

## Abbreviations

ASA, American Society of Anesthesiologists; BIS, Bispectral Index; CIs, confidence intervals; CONSORT, Consolidated Standards of Reporting Trials; ED_95_, 95% effective dose; ED_50_, median effective dose; HR, heart rate; NMDA, N-methyl-D-aspartate; RSS, Ramsay Sedation Scale; SpO_2_, pulse oxygen saturation; SBP, systolic blood pressure; SD, standard deviation; VAS, visual analog scores.
